# Sociodemographic Factors and Childhood Growth: Associations with Environmental Sanitation Phases

**DOI:** 10.3390/ijerph23010128

**Published:** 2026-01-20

**Authors:** Yadira Morejón-Terán, Ana Clara P. Campos, Juan Marcos Parise-Vasco, Leila Denise A. F. Amorim, Laura C. Rodrigues, Mauricio L. Barreto, Sheila Maria Alvim de Matos

**Affiliations:** 1 Programa de Pós-Graduação em Saúde Coletiva, Instituto de Saúde Coletiva, Universidade Federal da Bahia, Salvador 40110-040, BA, Brazil; ymorejon@hotmail.com (Y.M.-T.); acpcampos27@gmail.com (A.C.P.C.); mauricio@ufba.br (M.L.B.); sheilaalvim@gmail.com (S.M.A.d.M.); 2 Facultad de Ciencias de la Salud y Bienestar Humano, Universidad Tecnológica Indoamérica, Quito 170103, Ecuador; 3 Facultad de Salud y Bienestar, Pontificia Universidad Católica del Ecuador, Quito 170525, Ecuador; 4 Instituto de Matemática e Estatística, Universidad Federal de Bahia, Salvador 40170-110, BA, Brazil; leila.d.amorim1@gmail.com; 5 School of Hygiene and Tropical Medicine, Department of Infectious Disease Epidemiology, London WC1E 7HT, UK; laurarodriguesinlondon@gmail.com

**Keywords:** Anthropometric indicators, childhood, adolescent, environmental sanitation

## Abstract

**Highlights:**

**Public health relevance**

- Large-scale sanitation infrastructure programs affect multiple health outcomes simultaneously, making it critical to understand their long-term impact on child anthropometric indicators alongside changes in socioeconomic conditions.
- Early childhood growth trajectories can influence the risk of chronic diseases in adulthood, and understanding the socioeconomic factors that shape these trajectories is essential for addressing health inequalities during the critical first 1000 days of life.

**Public health significance**

- Children born in later phases of sanitation implementation showed improved linear growth trajectories (HAZ), particularly males, suggesting that sustained multi-level interventions can influence child development patterns over extended periods.
- Birth weight, household overcrowding, and maternal education emerged as consistent predictors of height-for-age across all sanitation phases, identifying modifiable targets for early childhood interventions in vulnerable populations.

**Public health implications**

- Interventions addressing modifiable factors such as household overcrowding and birth weight optimization may complement infrastructure improvements in promoting child growth in low-resource settings.
- Future interventions should adopt multisectoral approaches combining sanitation infrastructure with targeted nutritional support and maternal education programs, as infrastructure alone shows heterogeneous effects on growth outcomes.

**Abstract:**

Background: Early childhood growth trajectories can influence the risk of chronic diseases in adulthood. Improvements in environmental sanitation may affect child development in low-resource settings. Objective: to examine the associations among socioeconomic factors with nutrition indicators, and trajectories of anthropometric indicators across three epidemiological cohorts that reflect different phases of environmental sanitation implementation. Methods: A longitudinal study was conducted in Salvador, Brazil, from 1997 to 2013. A total of 1429 children were recruited across three epidemiological cohorts, corresponding to the phases of a sanitation program: pre-intervention (*n =* 299), intervention (*n =* 1007), and post-intervention (*n =* 123). Height-for-age (HAZ) and BMI-for-age (BAZ) z-scores were assessed at four time points. Multilevel linear models were used to adjust for socioeconomic factors. Results: A total of 992 children (68.7%) completed follow-up. Post-intervention children showed improved HAZ trajectories, with sex-specific patterns that varied across cohorts. Birth weight is positively associated with HAZ across all cohorts (0.34–0.49 kg increase per z-score). Household overcrowding (>2 persons/room) is consistently associated with lower HAZ (−0.34 to −0.63 z-score reduction). Children who were never exclusively breastfed in the post-intervention phase had a higher BAZ (0.76 z-score increase). Caesarean delivery is associated with higher BAZ in the pre-intervention (0.23) and intervention (0.27) cohorts. Conclusions: Children born in later time periods showed better growth trajectories, which may reflect the combined effects of sanitation improvements, economic development, and other societal changes in Brazil during this period. Further research using experimental or quasi-experimental designs is needed to isolate the specific contribution of sanitation to child growth.

## 1. Introduction

Child growth and development indicators in Brazil have shown substantial improvements over recent decades. The prevalence of stunting and undernutrition in children has decreased significantly since 1974, largely attributable to improvements in income, women’s education, access to medical care, economic development, safe water and sanitation [1]. These positive trends coincide with Brazil’s implementation of comprehensive social programs, including monetary transfers, improvements in sanitation systems, food security initiatives, expanded access to vaccines, and preventive health services to combat chronic and acute malnutrition [2].

Despite these improvements, significant social inequality and ethnic disparities persist throughout Brazil. Vulnerable populations, including blacks or browns, women, and youths, continue to report poorer health status, with increased risks of adverse outcomes such as low birth weight and obesity across the life cycle [3]. This ongoing disparity highlights the critical importance of understanding how social determinants shape physical growth trajectories from early childhood through adolescence.

The relationship between socioeconomic factors, environmental conditions, and children’s nutritional status has profound implications for addressing health inequalities during the first 1000 days [4]. Historically, malnutrition has been investigated and addressed from the perspectives of the impact of food insecurity on acute malnutrition, stunting, low birth weight and micronutrient deficiency; however, today, it encompasses other problems such as overweight and obesity [5]. Stunting is a growth disorder caused by undernutrition and poor health in the prenatal and postnatal periods. It can be associated with chronic nutrient deficiency or chronic or recurrent infectious disease. Factors associated with stunting include family socioeconomic status, parental education, mother’s nutritional status, birth weight, childhood illnesses, access to drinking water and sanitation services, and ethnic origin [6].

Intervention programs focused on infrastructure and sanitation improvements have shown little to no impact on children’s linear growth [7,8,9]. However, comprehensive interventions that include nutritional support are likely to have a greater effect [10]. Some intervention studies have been conducted at the scale of one or a few small communities. Large-scale sanitation programs pose significant challenges for evaluation and implementation; however, they can affect the transmission of various diseases in both public and private settings. The Bahia Azul Environmental Sanitation Program (Bahia-Azul) was the first program implemented at a large scale by the state of Salvador, Brazil, to quantify the effect of a sanitation program implemented throughout the city, three longitudinal epidemiological studies were established, which assessed the incidence and prevalence of diarrhoea in preschool children, nutritional status, and the prevalence and incidence of intestinal parasites, as well as sanitary and environmental conditions. This study showed that such large-scale interventions can offer widespread protection, with a 22% reduction in the longitudinal prevalence of diarrhoea and a 43% reduction in areas with higher baseline prevalence [11,12,13]. Given the importance of this study, the present study aimed to examine the associations among socioeconomic factors with nutrition indicators, and trajectories of anthropometric indicators across three epidemiological cohorts that reflect different phases of environmental sanitation implementation.

## 2. Materials and Methods

### 2.1. Study Design

A longitudinal study was conducted from childhood (0 to 5 years) to adolescence (12 to 19 years) in the metropolitan region of Salvador, Bahia, Brazil. This study compares three distinct epidemiological cohorts, recruited at different phases of the state sanitation program and subsequently followed into adolescence in an asthma study. It is important to note that this cross-cohort comparison design cannot establish causal relationships between sanitation improvements and growth outcomes, as the cohorts were born in different calendar years and thus experienced different secular conditions beyond sanitation changes.

### 2.2. Population and Sample

#### 2.2.1. The Bahia Azul Environmental Sanitation Program

**Figure 1:**
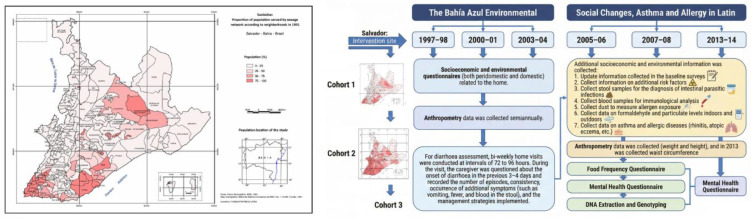
Study and information collected overview. Area according to sanitation proportion in the neighbourhoods of Salvador. Census 1991 and 2001 [16]. For the three epidemiological cohorts in the environmental sanitation program, protocols were standardised, and questionnaires were administered by a field coordinator (epidemiologist) and 15 interviewers over an extended follow-up period of 8 months or more to determine the prevalence and incidence of diarrhoea and parasites. For the SCAALA study, additional information on asthma and allergies was collected. The thick vertical lines indicate the cohorts by environmental sanitation phase and the progress of the health intervention.

Salvador is the capital of the State of Bahia, Brazil, with approximately 2.2 million inhabitants as of 1996 [14]. The Bahia Azul Environmental Sanitation Program (Bahia-Azul) was a large-scale state program aimed at increasing sanitation coverage from 26% to 80% of the population, with an investment of USD 440 million. The intervention included improvements to water supply and solid waste management, as well as strengthening institutional capacity. Approximately 2000 km of sewer pipes were installed, 86 pumping stations were constructed, and more than 300,000 households were connected to the sanitation network. Most household connections were completed in the project’s later years. Three million dollars were invested in public education campaigns focused on promoting sewer connections and responsible use of the system; the project did not include topics related to domestic hygiene promotion (Figure 1) [11,12,15,16].

#### 2.2.2. Original Epidemiological Cohorts

To evaluate the health impacts of the sanitation program, three separate epidemiological cohorts were established, each recruiting children aged 0–60 months during different phases of program implementation. Twenty-four sentinel areas were selected from 8 of the 18 drainage basins that would benefit from the Bahia Azul Program, corresponding to the poorest sectors of the city. These areas were classified according to census data as: low family income and predominantly healthy sanitation; low and moderately healthy family income; or low family income and unsanitised [16].

Approximately 20,000 households were enumerated in the selected sentinel areas, and those with children under five years were identified. From this sampling frame, households were randomly selected, and one child within the eligible age range was enrolled per household. This sampling strategy was designed to capture the population most likely to benefit from sanitation improvements, rather than to achieve population-level representativeness for Salvador as a whole.

**Figure 2:**
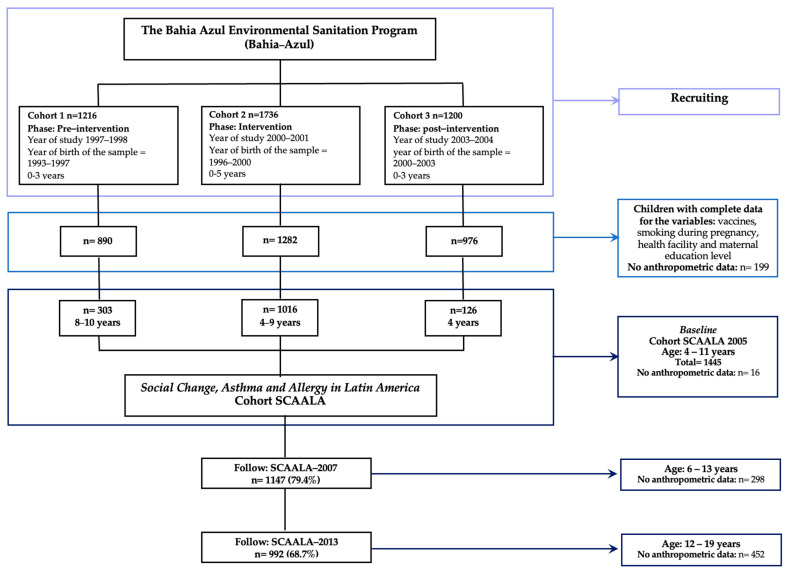
Flowchart of the study design, participants, and anthropometric data are available. Pre–intervention phase (baseline): 1997–1998 was defined as the first year, 2005–2006 (9th year), 2007–2008 (11th year), and 2013–2014 (17th year). Intervention phase (baseline): 2000–2001 (1st year), 2005–2006 (6th year), 2007–2008 (8th year), and 2013–2014 (14th year). Post–intervention phase (baseline): 2003–2004 (1st year), 2005–2005 (3rd year), 2007–2008 (5th year), and 2013–2014 (11th year).

The three cohorts were recruited as follows: pre-intervention cohort (December 1997 to December 1998, *n =* 1216 children); intervention cohort (October 2000 to January 2002, *n =* 1736 children); and post-intervention cohort (October 2003 to May 2004, *n =* 1200 children), totalling 4152 children aged 0–5 years (Figure 2) [11,12,15,17].

#### 2.2.3. Data Collection in Original Cohorts

For each epidemiological cohort, standardised protocols were implemented by a field coordinator (epidemiologist) and 15 trained interviewers. At baseline, individual and household questionnaires were administered to mothers or caregivers, collecting information on socioeconomic status, household living conditions, sanitation access, and child-related variables. Anthropometric measurements (weight and length/height) were collected semi-annually. For diarrhoea assessment, bi-weekly home visits were conducted at intervals of 72 to 96 h, during which caregivers were questioned about the onset of diarrhoea in the previous 3–4 days. The follow-up period for each original cohort extended 8 months or more to determine the prevalence and incidence of diarrhoea and intestinal parasites (Figure 1) [11,12,15,17].

#### 2.2.4. The SCAALA Cohort

In 2005, a new study was designed to investigate risk factors for asthma and other allergic diseases, building upon the detailed early-life exposure data collected in the original cohorts. Children from the three original cohorts who were aged 4 to 11 years in 2005 were eligible for inclusion if they had complete information on key variables including measles vaccination, maternal smoking during pregnancy, access to sanitation, maternal education, and stool examination results.

Sample size was calculated assuming a 10% prevalence of asthma, a 5% significance level, a 10% anticipated loss rate, and 80% statistical power, based on expected proportions of children exposed to specific risk factors. This calculation determined that 1445 children would be sufficient to detect meaningful associations with asthma outcomes, which was the primary objective of the SCAALA study. From the pool of eligible children meeting the inclusion criteria, a random sample of 1445 children were selected; this cohort was named the Social Changes, Asthma and Allergy in Latin America (SCAALA) study (Figure 2) [18].

Two additional follow-up assessments were conducted in 2007 and 2013, during which anthropometric measurements and questionnaires on asthma, allergies, and mental health were collected (Figure 1). An overview of the timeline and data collection is provided in Figure 1, including a description of questionnaires and periodicity for visits.

### 2.3. Variables and Data Source

#### 2.3.1. Anthropometric Measurements

The participants were weighed on portable microelectronic scales (model E-150/3P, Filizola Balanças Industriais, São Paulo, Brazil) with a capacity of up to 300 kg and a precision of 100 g. A wooden infantometer was used for length, and a stadiometer (Leicester Height Measure, Seca, Hamburg, Germany) was used for height. The instruments were calibrated periodically and were replaced with inelastic tape measures when necessary. Weight and length/height were measured twice; a 100 g and 0.1 cm variation between each measurement was accepted. The mean of the two measurements was considered the final measurement at each visit. The standards and techniques established by Lohman et al. (1988) [19] were applied to all anthropometric evaluations by a trained professional dietician. For each round, the children were weighed and measured, and different researchers took two series of measurements. Using strategic locations in each sentinel area (school, church, children’s club or residents’ association headquarters). The exact age in days was calculated by the difference between the date of birth and the date of measurement; this age was used to calculate age-specific z-scores.

The indicators were calculated in the Anthro Plus program V3.2.2 [20]. Outliers were detected using reports in the Anthro Plus; additionally, to ascertain the prevalence, the indicators were categorised using the cut-off points suggested by the World Health Organisation (WHO); valid values were considered Heigh-for-age z-score (HAZ) between −6 and +6 and Body Mass Index-for-age z-score (BAZ) between −5 and +5.

#### 2.3.2. Follow-Up Variable

The time variable was defined as the number of years of follow-up for each epidemiological cohort. The pre-intervention cohort was defined as 1997 (1st year), 2005 (9th year), 2007 (11th year), and 2013 (17th year). The intervention cohort was defined as 2000 (1st year), 2005 (6th year), 2007 (8th year), and 2013 (14th year). The post-intervention cohort was defined as 2003 (1st year), 2005 (3rd year), 2007 (5th year), and 2013 (11th year).

#### 2.3.3. Covariates

At the beginning of the three cohort studies, individual and household questionnaires were administered by mothers or caregivers, which included socioeconomic status, household living and sanitation conditions, and child-related variables. For this study, birth weight, Exclusive Breastfeeding, Overcrowding, mother’s education, mother’s ethnicity, and Smoking during the 1st year were considered as theoretical variables.

### 2.4. Statistical Analysis

The outcome variables were normally distributed, and no outliers were identified. To model growth trajectories across the study period in each cohort, we employed multilevel linear regression models to account for the hierarchical data structure (repeated measures nested within individuals). Initial analyses revealed high intraclass correlation coefficients for both HAZ (68%) and BAZ (61%), confirming the appropriateness of multilevel modelling, and statistical assumptions were analysed before the analysis.

Two multilevel linear model approaches were used for the trajectories in each cohort: (1) a quadratic time model with random intercept only and (2) a quadratic time model with random intercept and random slope for the time variable in each cohort, using an exchangeable correlation structure, selected after comparative evaluation of different covariance patterns. This choice was based on both theoretical foundations and the study design, as well as statistical criteria [21,22]. The selection of the best model was based on the Bayesian information criterion (BIC) and the Akaike Information Criterion (AIC) [23] (Supplementary Tables S3 and S5). The time variable was defined as the years of follow-up for each cohort phase of the sanitation program.

In addition, the following covariates (theoretical variables) were included in the multivariate linear model: type of delivery, birth weight, mother’s ethnic self-identification, exclusive breastfeeding, overcrowding (number of people per room), maternal education, and exposure to tobacco smoke during the first year of the child’s life.

For all outcomes and cohorts, the best model was the multilevel quadratic follow-up model with a time-squared term and a random intercept only. After evaluation of the data dependence structure, the covariates included in the multilevel model were: Birth weight (kg), Exclusive Breastfeeding, Overcrowding, Mother’s schooling, Mother’s ethnic self-identification, and Smoking during the 1st year. Means ± SD of estimated trajectories were also presented, stratified by gender, for HAZ and BAZ. Multilevel models have the advantage of being applicable under relatively weak assumptions about the missing-data mechanism, and potential bias is avoided with maximum-likelihood inference under any mechanism, except when the mechanism is not random. The models with a quadratic time model with random intercept and random slope for the time variable in each cohort are presented in Supplementary Tables S2 and S4, and the Akaike Information Criterion (AIC) and the Bayesian Information Criterion (BIC) results are presented in Supplementary Tables S3 and S5.

The final model was evaluated by analysing the distribution of standardised residuals. The normal distribution of the residuals was assessed using a quantile-quantile plot (Q-Q plot), and homoscedasticity was evaluated by plotting residuals versus fitted values. All statistical analyses were conducted using STATA software, version 17 [24].

### 2.5. Ethical Aspects

Each cohort epidemiological study was conducted in accordance with the Declaration of Helsinki. All study procedures were approved by the Ethics Committee of the Institute of Collective Health at the Federal University of Bahia (registration number 003-05/CEP-ISC and CEP/ISC 060-12). Participation was voluntary and contingent upon informed consent from the children’s guardians. The consent process included a detailed explanation of the study objectives and procedures. Written informed consent was documented for all participants.

## 3. Results

### 3.1. Participant Characteristics

Of the 1445 children recruited across the three epidemiological cohorts, anthropometric data were available for 992 (68.7%) participants at the final follow-up in 2013 (Figure 2). Males constituted more than 50% of the population across all three sanitation phases. Table 1 presents the sociodemographic characteristics of the study population by sanitation phase.

The first cohort had higher frequencies of birth weight greater than 3500 g, exclusive breastfeeding for less than four months, household overcrowding with more than two people per room, mothers with education ranging from illiterate to complete primary school, smoking during pregnancy, and exposure to tobacco smoke during the first year of life. The percentage of children in the second cohort who never received exclusive breastfeeding was 55.7%, and the percentage of Caesarean deliveries was highest in the third cohort (32.0%) (Table 1).

### 3.2. Nutritional Status Trends

A decrease in the percentage of “very low height for age” and “low height for age” was observed across all cohorts throughout the follow-up period. At the same time, the percentage of obesity increased: in the first cohort, by 0.9 percentage points (p.p.); in the second cohort, by 2.3 p.p.; and in the third cohort, by 2.9 p.p. (Supplementary Table S1).

### 3.3. Height-for-Age Z-Score Trajectory Analysis

An increase in the mean HAZ z-scores was observed between cohorts in relation to birth weight; for each kilogram, the mean z-scores were 1st cohort 0.34 (95% CI 0.15; 0.52), 2nd cohort 0.49 (95% CI 0.38; 0.61) and 3rd 0.43 (95% CI 0.05; 0.80); p. Overcrowding showed a reduction in mean z-scores for all cohorts: −0.38 (95% CI −0.66; −0.11), −0.34 (95% CI −0.51; −0.17), and −0.63 (95% CI −1.23; −0.03). Exposure to tobacco smoke during the first year of life showed a reduction in the mean z-score of −0.27 (95% CI −0.47; −0.08) in the third cohort (Table 2).

**Figure 3:**
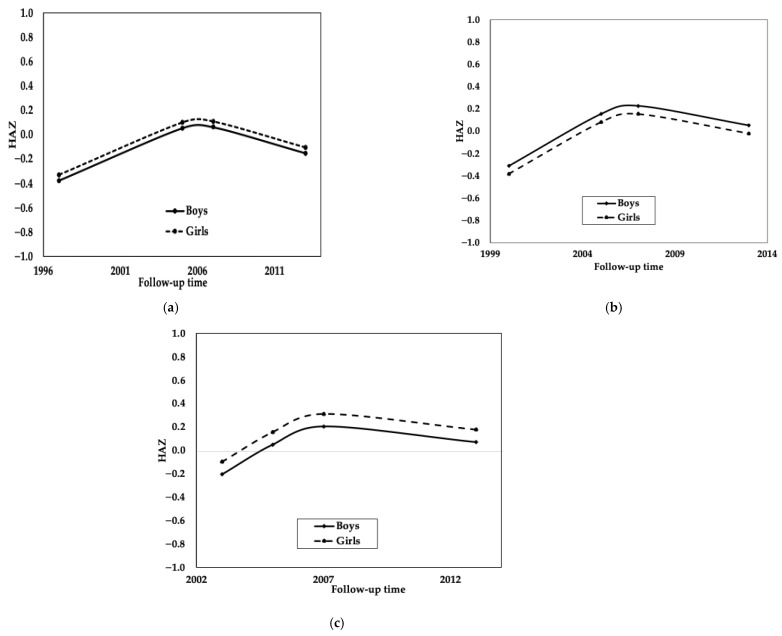
Mean Trajectories of Height-Age z-score (HAZ) by sex. The mean trajectory for the three cohorts was estimated using a quadratic time model with random intercept only. Legend: (**a**) HAZ 1997–2013, (**b**) HAZ 2000–2013, and (**c**) HAZ 2004–2013, and solid line is for boys and dotted line is for girls, Salvador-Bahia.

Figure 3 shows the predicted growth trajectories for HAZ z-scores by sex, using the results of a quadratic time model with random intercept only. These trajectories shift upwards until the third measurement and show a slight decline in the fourth measurement across all cohorts, consistent with typical patterns of linear growth deceleration as children approach final adult height.

The pattern of sex differences varied across cohorts. In the pre-intervention cohort, boys showed slightly higher mean HAZ scores than girls at the final measurement in 2013 (0.15 vs. 0.10, respectively). However, in the post-intervention cohort, this pattern was reversed, with girls showing higher mean scores than boys (0.17 vs. 0.07, respectively). In the intervention cohort, trajectories for both sexes followed similar patterns, with boys and girls converging toward comparable values by adolescence.

### 3.4. Body Mass Index-for-Age Z-Score Trajectory Analysis

An increase in mean HAZ z-scores was observed in the results of the pre-intervention and intervention cohorts in relation to birth weight; that is, for each kilogram, the mean z-scores were 0.35 (95% CI 0.12, 0.59) for the first cohort and 0.49 (95% CI 0.37, 0.62) for the second cohort. Caesarean delivery also showed an increase in mean z-scores: 0.23 (95% CI 0.07, 0.38) for the first cohort and 0.27 (95% CI 0.11, 0.42) for the second. Regarding breastfeeding, the mean z-scores increased in the third post-intervention cohort among children who had never been breastfed, to 0.76 (95% CI 0.22, 1.29) (Table 3).

**Figure 4:**
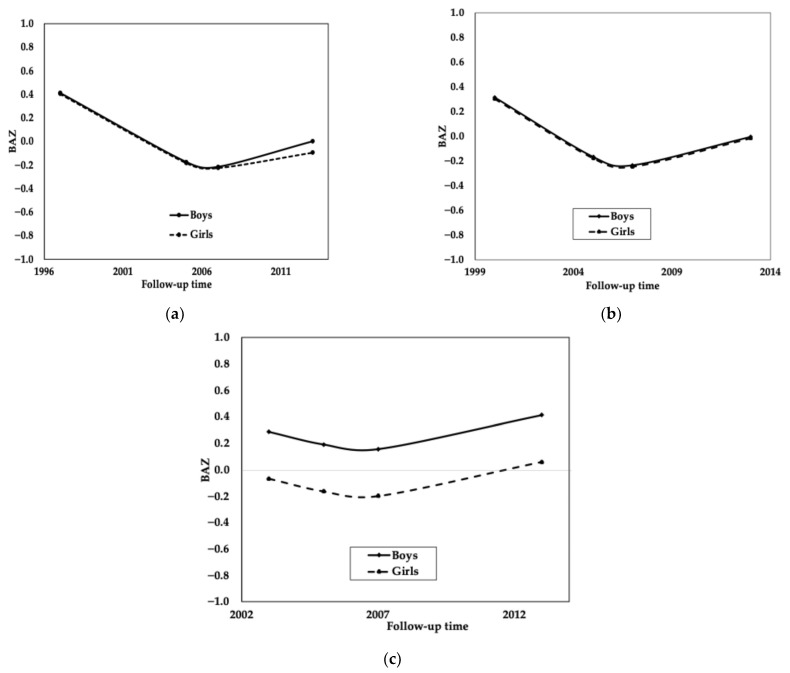
Mean Trajectories of Body-Mass-Index-Age z-score (BAZ) by sex. The mean trajectory for the three cohorts was estimated using a quadratic time model with random intercept only. Legend: (**a**) BAZ 1997–2013, (**b**) BAZ 2000–2013, and (**c**) BAZ 2004–2013, and the solid line is for boys, and the dotted line is for girls, Salvador-Bahia.

Figure 4 shows the projected growth trajectories for BAZ z-scores by sex, using the results of a quadratic time model with random intercept only. These trajectories show a decline in the first and second cohorts with a slight rebound in the fourth measurement. However, the post-intervention cohort showed a positive mean z-score of 0.41 for boys and 0.05 for girls in 2013, while the pre-intervention cohort started with mean z-scores of 0.41 for boys and 0.40 for girls and ended in adolescence with mean z-scores of −0.08 for boys and −0.09 for girls.

## 4. Discussion

This longitudinal study examined the association between socioeconomic factors and nutritional indicators of HAZ and BAZ in trajectories from childhood to adolescence across three epidemiological cohorts reflecting different stages of a large-scale state sanitation programme in Salvador, Brazil. These analyses showed associations between birth weight, maternal education, and overcrowding for HAZ, while for BAZ, birth weight and type of birth were associated in the pre-intervention and intervention cohorts; regarding exclusive breastfeeding, a significant association was observed in the post-intervention cohort. However, methodological limitations preclude establishing causal relationships between sanitation and the indicators’ outcomes, as the three cohorts were evaluated at different times during the programmes.

Previous evaluations of the Bahia Azul sanitation programme documented reductions in diarrhoeal episodes (43%) and intestinal parasite prevalence between the pre-intervention and post-intervention phases, including reductions in A. lumbricoides (42%), G. duodenalis (59%), and T. trichiura (62%) [11]. While these improvements in infectious disease outcomes occurred during the same period as our cohort recruitment, our study design does not permit direct linkage between these documented health improvements and the growth patterns observed in the present analysis. Other cohort studies, such as MAL-ED, have shown associations between biomarkers of environmental enteric dysfunction and child growth [25]; however, the pathways linking sanitation improvements to growth are complex, and effects of environmental interventions on nutritional indicators are often heterogeneous and attenuated by concurrent factors. In other words, despite changes in the health and sanitation environment of children in Salvador concerning diarrhoeal and parasitic diseases, children born during different periods of the sanitation phases may experience simultaneous changes in social, economic, and public service policies that prevent isolating the specific effect of the intervention on the nutritional indicators of interest in this study.

Randomised clinical trials that include environmental sanitation interventions, either alone or combined with health promotion and nutritional interventions, have shown little or no effect on growth and Z-score. These studies concluded that environmental improvements do not increase exposure to environmental pathogens in a way that affects growth, except in groups with a high enteric load. Regarding combined nutrition and sanitation interventions, these demonstrated an effect on biomarkers of intestinal inflammation, with minimal impact on growth [7,10,26,27,28,29].

Regarding birth weight, it has been considered a key factor in the growth of the child population. Some studies show that children with low birth weight are more likely to have short stature for their age and to experience poorer long-term recovery [30,31]. While birth weight is positively associated with overweight or obesity at later ages [32,33,34,35], these results are consistent with those of our study. In our research, children who never breastfed and were born by caesarean section showed an increase in the mean z-scores of the indicators, which aligns with other studies [36,37,38]. The interaction between birth weight and breastfeeding has been examined in several birth cohorts; the Ribeirão Preto and San Luis [39,40] cohorts in Brazil demonstrated that children born by caesarean section had higher BAZ z-scores even after adjusting for maternal and socioeconomic variables. However, the Pelotas and Porto Alegre cohorts [41,42,43] showed that the association between caesarean section and obesity diminishes and becomes non-significant after adjustment for maternal and socioeconomic variables, suggesting that this association may be explained by residual confounding.

The trajectories of HAZ and BAZ showed their z-scores for the different cohorts, which is consistent with other studies [44]. Research conducted in Brazil indicates that poor households have a higher risk of stunting and a lower body mass index, reflecting the coexistence of undernutrition and overnutrition. Changes in policies since the 2000s in Brazil, such as conditional cash transfer programmes (Bolsa Família), improvements in access to health services, safe water and sanitation, and protection policies, have contributed to a reduction in the prevalence of stunting and acute malnutrition [45,46,47]. Multisectoral interventions have helped reduce the national prevalence from 37% to 7% between 1974 and 2007. There is a trend towards increased height in the Brazilian population. Studies have shown a steady increase in the average. Between 1952 and 1967, an increase of 1 cm was identified, and between 1967 and 1982, an increase of 2.4 cm [48]. A recent population-based study showed an increase of 1 cm in the average height of children born between 2008 and 2014 [49].

A fundamental limitation of this study is its quasi-experimental design, which compares three separate cohorts (1997, 2000, 2003) rather than following the same children longitudinally through sanitation changes. This design cannot isolate sanitation effects from concurrent secular trends, including economic growth, expansions of the healthcare system, implementation of nutritional programs, and changes in infant feeding practices that occurred in Brazil during this period. The observed associations between later sanitation phases and improved growth trajectories may reflect these broader temporal changes rather than sanitation improvements specifically. Future studies employing difference-in-differences or instrumental variable approaches would be needed to establish causal relationships.

Additionally, the unequal sample sizes across cohorts (pre-intervention *n =* 299, intervention *n =* 1007, post-intervention *n =* 123) represent a methodological limitation. These differences resulted from the age eligibility criteria for SCAALA (4–11 years in 2005), which differentially captured children from cohorts recruited at different time periods, rather than from a sampling strategy designed for between-cohort comparisons. The small sample size of the post-intervention cohort (*n =* 123) may limit statistical power to detect associations in this group, and wider confidence intervals observed for this cohort reflect this reduced precision. Future studies designed specifically to compare growth across intervention phases should consider stratified sampling to ensure adequate and balanced representation across comparison groups.

## 5. Conclusions

Anthropometric indicators reflect multiple concurrent influences on child development. While children born during later phases of the sanitation program showed better growth trajectories, these improvements coincided with substantial changes in healthcare practices, infant feeding patterns, and socioeconomic conditions in Brazil. The dramatic shifts in exclusive breastfeeding rates and increasing Caesarean delivery rates observed across our cohorts exemplify the broader societal transformations occurring during the study period. Given these multiple concurrent changes and our cross-cohort design, we cannot attribute observed growth differences specifically to sanitation improvements. Future studies with appropriate control groups and designs capable of isolating sanitation effects from secular trends are needed to establish causal relationships.

## Figures and Tables

**Table 1:** Socioeconomic characteristics by environmental sanitation program phase. Salvador-Bahia.

Covariate			Pre-Intervention Cohort		Intervention Cohort		Post-Intervention Cohort	
Variables		Category	n	%	n	%	n	%
		Total	299		1007		123	
**Sex**		Female	129	43.1	476	47.3	60	48.8
		Male	170	56.9	531	52.7	63	51.2
Birth weight *		≤3500 g	207	71.1	678	71.4	98	81.0
		>3500 g	84	28.9	272	28.6	23	19.0
**Age (year)**		1st measure (mean (min; max))	0.9	(0; 3)	1.4	(0; 4)	2.7	(2; 3)
		2nd measure (mean (min; max))	8.7	(8; 10)	6.0	(4; 9)	4.0	(4; 4)
		3rd measure (mean (min; max))	10.7	(10; 13)	8.3	(6; 11)	6.2	(5; 7)
		4th measure (mean (min; max))	16.6	(16; 18)	14.1	(12; 17)	12.0	(11; 13)
Height (cm) *		1st measure (mean (IC95%))	78.7	(77.7; 79.6)	80.73	(79.8; 81.7)	95.6	(94.8; 96.5)
		2nd measure (mean (IC95%))	133.7	(132.9; 134.5)	118.9	(118.3; 119.5)	106.0	(105.1; 106.9)
		3rd measure (mean (IC95%))	147.4	(147.4; 148.4)	132.6	(131.9; 133.3)	120.9	(119.7; 122.1)
		4th measure (mean (IC95%)))	169.1	(167.9; 170.3)	163.2	(162.6; 163.9)	155.4	(153.6; 157.1)
Exclusive Breastfeeding *		≥4 month	81	27.2	84	8.3	37	30.1
		Never	29	9.7	560	55.7	20	16.3
		<4 month	188	73.1	362	36.0	66	53.7
Type of birth *		Vaginal	237	79.5	772	77.2	83	68.0
		Caesarean delivery/forceps	61	20.5	228	22.8	39	32.0
Ethnic self-identification *		Not Blacks	21	7.0	108	10.7	12	9.8
		Blacks	278	93.0	898	89.3	111	90.2
Overcrowding *		1 person per room	99	33.1	337	34.0	41	33.3
		2 people per room	132	44.2	453	45.7	60	48.8
		more than two people per room	68	22.7	201	20.3	22	17.9
Monthly income *		up to R$300	120	47.4	441	52.2	53	50.9
		R$301.00 to R$600.00	79	31.2	263	31.1	36	34.6
		greater than R$600.00	54	21.3	142	16.8	15	14.4
Mother’s education *		2 full degree to full superior	53	17.8	189	18.8	36	29.3
		Incomplete gymnasium to incomplete 2nd grade	157	52.7	573	56.9	59	47.9
		Illiterate to complete primary school	88	29.5	246	24.4	28	22.8
Smoking in pregnancy *		Not	265	87.8	902	89.3	112	88.9
		Yes	37	12.3	108	10.7	14	11.1
Smoking during the 1st year *		Not	261	87.6	885	88.3	111	90.2
		Yes	37	12.4	117	11.7	12	9.8

* Variables contain missing data; R$: Brazilian reais.

**Table 2:** Estimation of the effect of socioeconomic variables on the three-cohort phase of environmental sanitation for HAZ. Salvador-Bahia.

Covariates	Category	Pre-Intervention Phase *		Intervention Phase *		Post-Intervention Phase *	
		Estimate	95% IC	Estimate	95% IC	Estimate	95% IC
**Follow up**		0.10	0.08; 0.12 ᵻ	0.15	0.13; 0.16 ᵻ	0.23	0.16; 0.30 ᵻ
**Follow up ^2^**		−0.004	−0.006; −0.003	−0.008	−0.009; −0.007ᵻ	−0.022	−0.030; 0.014
**Birth weight (kg)**		0.34	0.15; 0.52 ᵻ	0.49	0.38; 0.61 ᵻ	0.43	0.05; 0.80 ᵻ
**Exclusive Breastfeeding (≥4 months)**	Never	−0.10	−0.45; 0.23	−0.02	−0.17; 0.12	−0.11	−0.62; 0.38
	<4 months	0.26	0.05; 0.47 ᵻ	0.06	−0.11; 0.24	0.04	−0.41; 0.49
**Overcrowding** **(1 p/room)**	2 people per room	−0.09	−0.32; 0.12	−0.21	−0.34; −0.07 ᵻ	−0.43	−0.84; −0.03 ᵻ
	more than 2 people per room	−0.38	−0.66; −0.11 ᵻ	−0.34	−0.51; −0.17 ᵻ	−0.63	−1.23; −0.03 ᵻ
**Mother’s schooling (2 g.c. to s.c)**	Incomplete gymnasium to incomplete 2nd grade	0.05	−0.21; 0.32	−0.24	−0.40; −0.09 ᵻ	0.00	−0.43; 0.44
	Illiterate to complete primary school	−0.31	−0.61; −0.00 ᵻ	−0.23	−0.42; −0.04 ᵻ	−0.18	−0.72; 0.35
**Mother’s ethnic self-identification**	Black	0.23	−0.14; 0.60	0.08	−0.10; 0.27	−0.20	−0.81; 0.41
**Smoking during the 1st year (No)**		0.02	−0.27; 0.33	−0.27	−0.47; −0.08 ᵻ	−0.27	−0.41; 0.96
**Interclass Correlation Coefficient**		0.60		0.68		0.86	

* Linear model with random intercept only; ᵻ *p* < 0.05; ^2^ age quadratic variable.

**Table 3:** Estimation of the effect of socioeconomic variables on the three-cohort phase of environmental sanitation for BAZ. Salvador-Bahia.

Covariates	Category	Pre-Intervention Phase *		Intervention Phase *		Post-Intervention Phase *	
		Estimate	95% IC	Estimate	95% IC	Estimate	95% IC
**Follow up**		−0.12	−0.15; −0.09 ᵻ	−0.09	−0.19; −0.06	0.14	−0.25; −0.03 ᵻ
**Follow up ^2^**		0.004	0.003; 0.01 ᵻ	0.009	0.007; 0.01 ᵻ	0.018	0.005; 0.029 ᵻ
**Birth weight (kg)**		0.35	0.12; 0.59 ᵻ	0.49	0.37; 0.62 ᵻ	0.28	−0.10; 0.66
**Type of** **birth** **(vaginal)**	Caesarean delivery/Forceps	0.23	0.07; 0.38 ᵻ	0.27	0.11; 0.42 ᵻ	0.07	−0.12; 0.28
**Mother’s ethnic self-identification**	Black	−0.18	−0.65; 0.29	−0.16	−0.36; 0.04	−0.49	−1.13; 0.13
**Exclusive Breastfeeding (≥4 months)**	Never	−0.09	−0.53; 0.34	−0.11	−0.28; 0.05	0.76	0.22; 1.29 ᵻ
	<4 months	−0.04	−0.31; 0.22	−0.01	−0.20; 0.17	0.24	−0.22; 0.70
**Overcrowding (1 p/room)**	2 people per room	−0.11	−0.40; 0.16	−0.10	−0.24; 0.04	−0.39	−0.22; 0.70
	more than 2 people per room	−0.32	−0.68; 0.02	−0.29	−0.48; −0.10 ᵻ	−0.58	−1.18; 0.00
**Mother’s schooling (2 g.c. to s.c)**	Incomplete gymnasium to incomplete 2nd grade	−0.02	−0.36; 0.31	−0.07	−0.24; 0.09	−0.25	−0.70; 0.19
	Illiterate to complete primary school	−0.15	−0.53; 0.23	−0.13	−0.34; 0.06	−0.38	−0.93; 0.16
**Interclass Correlation Coefficient**		0.62		0.68		0.77	

* Linear model with random intercept only; ᵻ *p* < 0.05; ^2^ age quadratic variable.

## Data Availability

The data from this study are part of two research projects conducted in Salvador de Bahia: Bahia Azul and SCAALA cohort, in which children from unsanitary areas participated.

## Supplementary Materials

The following supporting information can be downloaded at: https://www.mdpi.com/article/10.3390/ijerph23010128/s1, Table S1: Supplementary Table S1. Percentage of nutritional status according to the HAZ and BAZ indicators for all cohorts, Salvador-Bahia; Table S2: Estimation of the effect of socioeconomic variables according to the environmental sanitation phase A//E. Salvador- Bahia; Table S3: Goodness-of-fit statistical tests for (1) time-squared model with random intercept only and (2) time-squared model with random intercept and random slope for the time variable. HAZ. Salvador- Bahia; Table S4: Estimating the effect of socioeconomic variables according to the phase of environmental sanitation IMC//E. Salvador- Bahia; Table S5: Goodness-of-fit statistical tests for (1) time-squared model with random intercept only and (2) time-squared model with random intercept and random slope for the time variable. BMI//E. Salvador- Bahia.

## Abbreviations

BIC	Bayesian information criterion
HAZ	Height-for-age Z-score
NCDs	Non-communicable diseases
SCAALA	Social Change, Asthma, and Allergy in Latin America
WHO	World Health Organization
CEP-ISC	Comité de Ética en Pesquisa—Instituto de Salud Colectiva
CISPEC	Centro de Investigación en Salud Pública y Epidemiología Clínica
ISC/UFBA	Instituto de Salud Colectiva/Universidad Federal de Bahia
