# Supplementary material for: Sociodemographic Factors and Childhood Growth: Associations with Environmental Sanitation Phases
Source: Int J Environ Res Public Health. 2026 Jan 20;23(1):128. doi: 10.3390/ijerph23010128 (PMC12840589; doi:10.3390/ijerph23010128)
Supplement: Supplementary file 1 [file ijerph-23-00128-s001.zip › ijerph-3941665-supplementary.pdf]

Supplementary Table S1. Percentage of nutritional status according to the HAZ and BAZ indicators for all cohorts, Salvador-Bahia.

Anthropometric indicator	Category	Cohort 1: Pre-intervention phase							
		Bahía-Azul 1997 (1° year)		SCAALA 2005 (9° year)		SCAALA 2007 (11° year)		SCAALA 2013 (17° year)	
		n	%	n	%	n	%	n	%
Height-Age z-score (HAZ)	Very low for age	6	2.1	---	---	---	---	---	---
	Low height for age	12	4.1	6	2.1	4	2.0	1	0.5
	Appropriate height for age	275	93.8	293	97.9	242	97.8	195	99.5
Body-Mass-Index-for-age z-score (BAZ)	Underweight	1	0.3	12	4.0	19	7.7	5	2.6
	Normal	204	69.4	232	77.6	174	70.7	153	78.1
	Overweight	66	22.5	40	13.4	39	15.9	21	10.7
	Obesity	23	7.8	15	5.0	14	5.7	17	8.7
**Anthropometric indicator**	**Category**	**Cohort 2: Intervention phase**							
		**Bahía-Azul 2000 (1° year)**		**SCAALA 2005 (6° year)**		**SCAALA 2007 (8° year)**		**SCAALA 2013 (14° year)**	
		**n**	**%**	**n**	**%**	**n**	**%**	**n**	**%**
Height-Age z-score (HAZ)	Very low for age	11	1.25	5	0.5	2	0.3	1	0.1
	Low height for age	63	7.18	20	1.9	17	2.1	17	2.4
	Appropriate height for age	804	91.6	982	97.5	788	97.7	692	97.5
Body-Mass-Index-for-age z-score (BAZ)	Underweight	10	1.1	38	3.8	47	5.8	35	4.9
	Normal	653	74.5	835	82.9	609	75.5	525	73.9
	Overweight	164	18.7	88	8.7	99	12.3	93	13.1
	Obesity	50	5.7	46	4.6	52	6.4	57	8.0
**Anthropometric indicator**	**Category**	**Cohort 3: Post-intervention phase**							
		**Bahía-Azul 2003 (1° year)**		**SCAALA 2005 (3° year)**		**SCAALA 2007 (5° year)**		**SCAALA 2013 (11° year)**	
		**n**	**%**	**n**	**%**	**n**	**%**	**n**	**%**
Height-Age z-score (HAZ)	Very low for age	1	0.8	---	---	---	---	1	1.1
	Low height for age	7	5.7	4	3.3	2	2.1	1	1.1
	Appropriate height for age	115	93.5	119	96.8	92	97.8	86	97.7
Body-Mass-Index-for-age z-score (BAZ)	Underweight	2	1.6	---	---	3	3.2	4	4.6
	Normal	102	82.9	102	82.9	75	79.8	64	72.7
	Overweight	10	8.1	12	9.8	10	10.6	11	12.5
	Obesity	9	7.3	9	7.3	6	6.4	9	10.2

Supplementary Table S2. Estimation of the effect of socioeconomic variables on the three-cohort phase of environmental sanitation HAZ. Salvador- Bahia.

Covariates	Category	Pre-intervention phase*		Intervention phase*		Post-intervention phase*	
		Estimate	95% IC	Estimate	95% IC	Estimate	95% IC
**Follow up**		0.09	0.07; 0.12 ᵻ	0.14	0.12; 0.16 ᵻ	-0.43	-0.83; -0.03
**Follow up^2**		-0.004	-0.006; -0.003 ᵻ	-0.008	-0.009; -0.006 ᵻ	-0.043	-0.061; -0.025 ᵻ
**Birth weight (kg)**		0.41	0.28; 0.55 ᵻ	0.65	0.57; 0.73 ᵻ	0.42	0.20; 0.64 ᵻ
**Exclusive Breastfeeding (>= 4 months)**	*Never*	-0.12	-0.37; 0.11	0.10	0.01; 0.20	-0.14	-0.43; 0.15
	<4 months	0.28	0.13; 0.43 ᵻ	0.14	0.02; 0.26	0.01	-0.25; 0.28
**Overcrowding (1 p/room)**	*2 people per room*	-0.14	-0.30; 0.01	-0.27	-0.36; -0.18 ᵻ	-0.43	-0.67; -0.19 ᵻ
	more than 2 people per room	-0.45	-0.64; -0.25 ᵻ	-0.40	-0.52; -0.28 ᵻ	-0.80	-1.15; -0.45 ᵻ
**Maternal education (2 g.c. to s.c)**	*Incomplete gynasium to incomplete 2nd grade*	0.03	-0.15; 0.22	-0.25	-0.36; -0.14 ᵻ	-0.04	-0.30; 0.21
	Illiterate to complete primary school	-0.33	-0.55; -0.12 ᵻ	-0.23	-0.36; -0.09 ᵻ	-0.29	-0.60; 0.02
**Mother's ethnic self-identification**	*Black*	0.23	-0.03; 0.49	0.04	-0.07; 0.17	-0.16	-0.53; 0.20
**Smoking during the 1st year (No)**		0.05	-0.16; 0.26	-0.27	-0.40; -0.14 ᵻ	0.47	0.06; 0.87 ᵻ
**Interclass Correlation Coefficient**		0.60		0.68		0.86	

*linear model with random intercept and random slope for the time variable
ᵻ $p<0.05$

Supplementary Table S3. Goodness-of-fit statistical tests for 1) a quadratic time model with random intercept only and (2) a quadratic time model with random intercept and random slope for the time variable in each cohort. HAZ. Salvador- Bahia.

Statistics	Pre-intervention phase 1997	
	Intercept (1)	intercept and inclination (2)
AIC	2362.97	2644.96
BIC	2431.67	2718.56
	Intervention Phase 2000	
AIC	7458.64	8359.27
BIC	7543.41	8450.10
	Post-intervention phase 2003	
AIC	792.20	1094.40
BIC	848.83	1159.12

Supplementary Table S4. Estimation of the effect of socioeconomic variables on the three-cohort phase of environmental sanitation BAZ. Salvador- Bahia.

Covariates	Category	Pre-intervention phase*		Intervention phase*		Post-intervention phase*	
		Estimate	95% IC	Estimate	95% IC	Estimate	95% IC
**Follow up**		-0.11	-0.14; -0.08 ᵻ	-0.16	-0.17; -0.13 ᵻ	-0.14	-0.29; 0.00
**Follow up^2**		0.004	0.003; 0.006 ᵻ	0.009	0.007; 0.01 ᵻ	0.01	0.007; 0.03 ᵻ
**Birth weight (kg)**		0.38	0.22; 0.53 ᵻ	0.53	0.44; 0.61 ᵻ	0.34	0.09; 0.60ᵻ
**Type of birth (vaginal)**	*Forceps Caesarean delivery/Forceps*	0.20	0.10; 0.30 ᵻ	0.13	0.07; 0.18 ᵻ	0.04	-0.08; 0.18
**Mother's ethnic self-identification**	*Black*	-0.05	-0.36; 0.25	-0.09	-0.24; 0.04	-0.45	-0.88; -0.03 ᵻ
**Exclusive**	*Never*	-0.04	-0.33; 0.24	-0.10	-0.21; 0.00	0.54	0.19; 0.89 ᵻ
**Breastfeeding (>= 4 months)**	*<4 months*	-0.15	-0.32; 0.02	0.06	-0.06; 0.20	0.18	-0.12; 0.49
**Overcrowding (1 p/room)**	*2 people per room*	-0.18	-0.36; 0.00	-0.11	-0.22; -0.01 ᵻ	-0.43	-0.71; -0.15 ᵻ
	more than 2 people per room	-0.30	-0.53; -0.07 ᵻ	-0.22	-0.35; -0.08 ᵻ	-0.53	-0.92; -0.13 ᵻ
**Maternal education (2 g.c. to s.c)**	*Incomplete gynasium to incomplete 2nd grade*	-0.03	-0.25; 0.18	-0.01	-0.13; 0.10	-0.15	-0.45; 0.14
	Illiterate to complete primary school	-0.17	-0.42; 0.07	-0.07	-0.22; 0.06	-0.34	-0.71; 0.01
**Interclass Correlation Coefficient**		0.62		0.68		0.77	

*linear model with random intercept and random slope for the variable time

ᵻ $p<0.05$

Supplementary Table S5. Goodness-of-fit statistical tests for 1) a quadratic time model with random intercept only and (2) a quadratic time model with random intercept and random slope for the time variable in each cohort. BAZ. Salvador- Bahia.

Statistics	Pre-intervention phase 1997	
	Intercept (1)	intercept and inclination (2)
AIC	2750.16	2826.46
BIC	2818.80	2900.00
	Intervention Phase 2000	
AIC	8727.04	8934.05
BIC	8811.83	9024.89
	Post-intervention phase 2003	
AIC	1047.79	1211.44
BIC	1104.29	1271.97
